# *Special series on “The meaning of behavioral medicine in the psychosomatic field”* establishment of a core curriculum for behavioral science in Japan: The importance of such a curriculum from the perspective of psychology

**DOI:** 10.1186/s13030-016-0056-6

**Published:** 2016-03-02

**Authors:** Akihito Shimazu, Mutsuhiro Nakao

**Affiliations:** Department of Mental Health, the University of Tokyo Graduate School of Medicine, Tokyo, Japan; Teikyo University Graduate School of Public Health & Department of Psychosomatic Medicine, Teikyo University Hospital, 2-11-1, Kaga, Itabashi, Tokyo, 173-8605 Japan

**Keywords:** Behavioral science, Core curriculum, Psychology

## Abstract

This article discusses the core curriculum for behavioral science, from the perspective of psychology, recommended by the Japanese Society of Behavioral Medicine and seeks to explain how the curriculum can be effectively implemented in medical and health-related departments. First, the content of the core curriculum is reviewed from the perspective of psychology. We show that the curriculum features both basic and applied components and that the basic components are closely related to various aspects of psychology. Next, we emphasize two points to aid the effective delivery of the curriculum: 1) It is necessary to explain the purpose and significance of basic components of behavioral science to improve student motivation; and 2) it is important to encourage student self-efficacy to facilitate application of the acquired knowledge and skills in clinical practice.

## Background

Curricula in Japanese medical schools have changed rapidly to accommodate the requirements of the Educational Commission for Foreign Medical Graduates of the U.S.A. and the Basic Medical Education Global Standards for Quality Improvement, issued by the World Federation for Medical Education (WFME); this is the so-called “2023 problem”. The curricular changes are based on the Basic Medical Education: Japanese Specifications WFME Global Standards for Quality Improvement [1]. In the Japanese version of the basic medical education global standards, behavioral science is listed as a core item, together with basic and clinical medicine. However, it has been unusual to offer education in behavioral science as an independent curriculum item in Japanese medical schools, and a model core curriculum for education in behavioral science was therefore required.

Against such a background, the Japanese Society of Behavioral Medicine (JSBM) organized a working group in co-operation with the JSBM Committee of Education and Training. The working group was to establish a core curriculum in behavioral science/behavioral medicine to enhance medical education in Japan [2]. In this article, the proposed curriculum is reviewed from the perspective of psychology, and we emphasize two important points that will facilitate the effective implementation of the curriculum in medical and health-related departments. It is relevant to observe that the first author (A.S.) is a specialist in psychology. A.S. received training in psychology prior to award of the doctoral degree, has trained students to become clinical psychologists, and now works in mental health education and research in a graduate school of public health attached to a university medical school. The second author (M.N.) is a specialist in psychosomatic medicine. M.N. is a physician who teaches and researches behavioral medicine in a graduate school of public health and also clinically consults in psychosomatic medicine at the hospital attached to the medical school of the university.

## Core curriculum related to psychology

Psychology is the science of mind and behavior and spans all aspects of human life, from the individual to the group. The major study areas of psychology are listed in Table 1 [3]. Of these 27 major areas, 13 (48 %) were selected by the JSBM working group [4] and included in the JSBM core curriculum in behavioral science (Table 2).

**Table 1:** Major areas of study in psychology (27 items)

●principles and history	●research methods	●learning
●cognition	●perception	●development
●education	●social (psychology)	●emotion
●personality	●clinical (psychology)	●disorder
●nervous function	●physiology	●statistics
●measurement and assessment	●industry	●organization
●health	●welfare	●crime and justice
●delinquency	●evolution	●heredity
●environment	●culture	●behavioral economics

**Table 2:** Areas of psychology related to the JSBM core curriculum for behavioral science

Area of psychology	Context
Psychology of learning	The process of learning (acquisition of behavior) in humans and animals
Cognitive psychology	Mental processes: (for example) thinking, language, memory, and imaging.
Perceptual psychology	The sensory process and the use of perceptual organs to deal with external information
Developmental psychology	The laws of development; mind/body formation and growth
Social psychology	The general social behavior of humans
Personality psychology	The characteristics of personality, motivation, and individual differences
Clinical psychology	Psychological assistance and prevention of psychological and behavioral disorders
Neuropsychology	The relationship between the nervous system (especially the brain) and mental functioning (especially language and cognition)
Psychophysiology	The relationship between behavior and physiological mechanisms
Industrial and organizational psychology	The organizational behaviors of humans in the context of interactions between individuals and the surrounding organizational environment
Health psychology	Application of psychological knowledge in the management of chronic diseases, identification of behavioral factors influencing disease, disease prevention and rehabilitation, and the maintenance and promotion of health behaviors
Cultural psychology	The interactions between culture and human behaviors

The details of the curriculum are shown in Table 3. The curriculum features 15 sessions, consisting of 11 lectures and 4 exercises. Basic aspects of behavioral modification are taught in sessions 1–8. More specifically, the first session (“shaping”) introduces the learning theories of classical and operant conditioning. These are taught from the perspective of acquisition, maintenance, and extinction of behaviors. The second session (“motivation”) deals with how motivation serves to drive behavioral change in terms of initiation, continuation, and termination of behavior and also explores the origin and management of conflict. The third session (“psychological stress”) focuses on theories of stress arousal; dealing also with stressful life events and psychological stress models (stressors, cognitive appraisal, coping, and stress responses). Effective methods of stress management are also explained. The fourth session (“environmental stress and health”) focuses on life-related stresses such as job stress, school stress, and child care stress. Effective methods of management and support are introduced. In the fifth session (“life-span development”), the human life from birth to death is considered to be a process of development, and the psychological and behavioral characteristics of each developmental stage are presented from a life-cycle perspective. The sixth session (“individual differences”) deals mainly with among-individual differences in behaviors including personality, intelligence, and role theory. In the seventh session (“interpersonal relationships”), the associations of various behaviors in the context of interactions with others and activities in social situations are classified into self, interpersonal, group, and cultural associations. Factors that are either determinant of, or influence, each behavior are explained. The eighth session (“theories of behavior modification”) deals principally with behavioral therapy. Cognitive behavioral therapy is presented as the theoretical framework facilitating behavioral modification in humans.

**Table 3:** The JSBM core curriculum for behavioral science and its association with psychological topics

Lecture theme	Lecture keywords and training methods	Related areas of psychology
1. Shaping	• Imprinting • Classical (respondent) conditioning • Operant (instrumental) conditioning • Cognitive learning • Social learning (observation and imitation) • Brain neurotransmitters	Learning, cognition, nervous function, physiology, society
2. Motivation	• Motivation (intrinsic and extrinsic) • Needs • Frustration • Conflict • Adjustment mechanisms • Defense mechanisms	Education, practice, health
3. Stress (psychological)	• Stressors • Stress reactions • Psychological models of stress • Cognitive appraisal • Coping • Life events • Relaxation techniques	Practice, physiology, health
4. Stress (environmental) and health	• Workplace stress • Other kinds of stress (childhood stress, parenting stress, etc.) • Stress management • Social support	Practice, development, industry, organization, health
5. Life-span development	• Mental development • Life cycle • Gene-environment interaction • Life tasks	Development, heredity
6. Individual differences	• Personality • Type theory • Trait theory • The Big Five • Intelligence • Role theory • Gender	Personality, development, society
7. Interpersonal relationships	• Interpersonal cognition • Needs and conflict • Group dynamics • Social adjustment • Interpersonal communication • Culture	Society, culture
8. Theories of behavioral modification	• Motivation • Behavioral therapy • Cognitive behavioral therapy • Stimulus control • Self-efficacy • Multiple-theory integration model • Empowerment	Learning, cognition, practice, health
9. Behavior modification techniques	• Lifestyle guidance • Health guidance (smoking cessation/medication/counseling) • Teaching and coaching	Learning, cognition, education, practice, health
10. Health communication	• Dissemination of public health and medical information (guidelines, participation in medical examinations) • Doctor-patient communication • Communication between medical practitioners	Society, health
11. Society and health	• Inequality and health • Social capital • Social participation • Social epidemiology • Social determinants of health • Cultural competence	Society, health, culture
12–15. Seminars/ practicums	Proposals for actual treatment strategies and role-plays based on medical scenarios	Practice, health

Applications in medicine and health settings are dealt with in sessions 9–11, after sessions 1–8 (on basic content) have been completed. More specifically, in session 9 (“behavior modification techniques”), a variety of methods used to modify inappropriate lifestyles are introduced. Session 10 (“health communication”) deals with how public health and medical information is disseminated and examines doctor-patient communication and communication between medical practitioners. Session 11 (“society and health”) deals with determinants of human health from a macro perspective; solutions and strategies (including health policies) are introduced. The remaining sessions (12–15) feature discussion on actual treatment strategies using simulated cases, role-play, and applications in clinical practice.

Sessions 5–8 of the curriculum deal principally with basic theories on the human mind and behavior; these are commonly taught in most psychology departments. In contrast, lecture sessions 9–11 and practice sessions 12–15 focus on medical applications of the basic theories mastered in the preceding sessions, which are not necessarily taught in departments of psychology.

## Conclusions

### Toward effective implementation of the core curriculum

In this article, we review the JSBM core curriculum in behavioral science from the perspective of psychology. We find that the curriculum can be classified into basic components (sessions 1–8) and practical components (sessions 9–11 and 12–15). The basic topics are closely related to broad areas of psychology, and the practical components are offered in medical schools and departments responsible for health and medical care. Based on our findings, we emphasize the following two points; our aim was to ensure that the proposed JSBM curriculum operates effectively in medical schools and in departments responsible for health and medical care.

First, it is important for students to be told why the basic content (the theory) is important; this increases the motivation to learn. There are at least three reasons why medical and health professionals should learn behavioral theories relevant to health: 1. Such professionals need to logically consider how to modify and maintain health-related behavior; 2. The professionals need to discuss modifications and maintenance of treatment with colleagues using a “common language”; and, 3. The professionals must understand the current conditions of targeted patients, make appropriate plans, take appropriate action, and schedule appropriate assessments by applying behavioral theories [5]. Medical students and others studying at departments responsible for health and medical care are more likely to have clear views of their future career paths than do other students. Therefore, efforts must be made to motivate such students by introducing actual cases and sharing all available knowledge when basic components are introduced.

Second, it is important that students maximize their motivation in order to facilitate the use, in real practice, of the knowledge and skills acquired during study sessions. It is not enough simply to learn; the newly acquired skills must be used in the clinic. The sessions employ case studies and role-play to emphasize the importance of the terms “enable” and “utilize”. Again, it is not enough to “simply understand” the content of the curriculum; it is essential to enhance self-efficacy. To motivate students, it is important for psychosomatic medicine practitioners to actively collaborate in the curriculum of behavioral medicine, especially in the practical components of sessions 9–15.

The JSBM working group on the core curriculum in behavioral science concedes that the incorporation of aspects of “cultural diversity” and “cultural competence” into the curriculum remains a work-in-progress. This is often the case when establishing a curriculum in medical education [6]. The working group is multidisciplinary, and the members plan to continue to discuss these topics. Psychologists play indispensable roles in such discussions, working with experts in psychosomatic medicine.
